# Hormonal Regulation of Stem Cell Proliferation at the *Arabidopsis thaliana* Root Stem Cell Niche

**DOI:** 10.3389/fpls.2021.628491

**Published:** 2021-03-03

**Authors:** Mónica L. García-Gómez, Adriana Garay-Arroyo, Berenice García-Ponce, María de la Paz Sánchez, Elena R. Álvarez-Buylla

**Affiliations:** ^1^Laboratorio de Genética Molecular, Desarrollo y Evolución de Plantas, Departamento de Ecología Funcional, Instituto de Ecología, Universidad Nacional Autónoma de México, Ciudad de México, Mexico; ^2^Centro de Ciencias de la Complejidad, Universidad Nacional Autónoma de México, Ciudad de México, Mexico

**Keywords:** root stem cell niche, quiescent center, stem cell regulation, gene regulatory networks, plant development, hormonal regulation

## Abstract

The root stem cell niche (SCN) of *Arabidopsis thaliana* consists of the quiescent center (QC) cells and the surrounding initial stem cells that produce progeny to replenish all the tissues of the root. The QC cells divide rather slowly relative to the initials, yet most root tissues can be formed from these cells, depending on the requirements of the plant. Hormones are fundamental cues that link such needs with the cell proliferation and differentiation dynamics at the root SCN. Nonetheless, the crosstalk between hormone signaling and the mechanisms that regulate developmental adjustments is still not fully understood. Developmental transcriptional regulatory networks modulate hormone biosynthesis, metabolism, and signaling, and conversely, hormonal responses can affect the expression of transcription factors involved in the spatiotemporal patterning at the root SCN. Hence, a complex genetic–hormonal regulatory network underlies root patterning, growth, and plasticity in response to changing environmental conditions. In this review, we summarize the scientific literature regarding the role of hormones in the regulation of QC cell proliferation and discuss how hormonal signaling pathways may be integrated with the gene regulatory network that underlies cell fate in the root SCN. The conceptual framework we present aims to contribute to the understanding of the mechanisms by which hormonal pathways act as integrators of environmental cues to impact on SCN activity.

## Introduction

**FIGURE 1:**
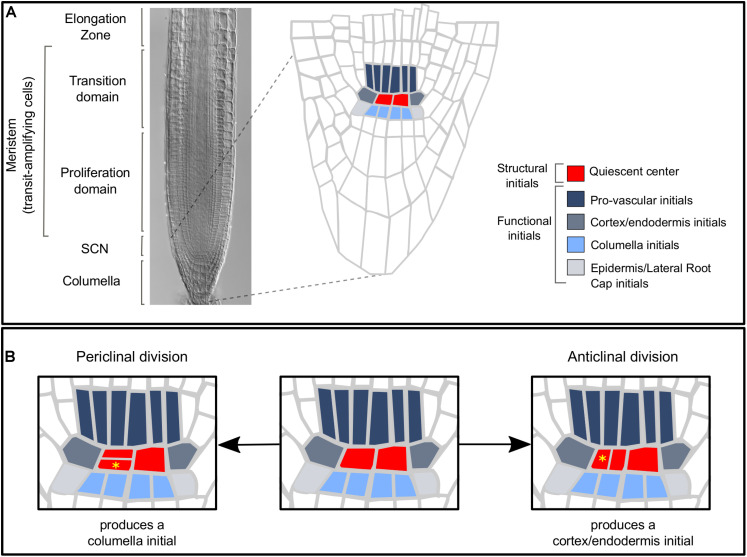
**(A)** The root apical meristem is composed of the SCN, the proliferation domain, and the transition domain (Ivanov and Dubrovsky, 2013). The SCN houses the QC cells and the ICs [structural and funcional initials, respectively (Barlow, 1997)] which divide asymmetrically and produce cells of different root tissues. The QC cells produce most root tissues and are considered a reserve of multipotent stem cells. **(B)** QC divisions can be periclinal or anticlinal and produce columella initials or cortex/endodermis initials, respectively. Yellow asterisks mark the daughter cells that replace an initial cell in each case.

Stem cells (SCs) are undifferentiated cells that can self-renew and produce progeny that replenishes and regenerates the tissues of multicellular organisms (Alvarado and Yamanaka, 2014). The root stem cell niche (SCN) of *Arabidopsis thaliana* (*Arabidopsis* hereafter) has a relatively simple structure, a stereotypical number of SCs, and a highly regular pattern of cell divisions (Dolan et al., 1993) (Figure 1A), making it a unique model to characterize the dynamics of SC activity in living organs. The SCN is located at the root apex and consists of the quiescent center (QC) and the stem or initial cells (ICs) (Barlow, 1978; Dolan et al., 1993; Barlow, 1997; Heidstra and Sabatini, 2014). Depending on their position relative to the QC, ICs produce cells that will become part of the different tissues of the root (Dolan et al., 1993) (Figure 1A). The cortex/endodermis initials, the provascular initials, and the epidermis and lateral root cap initials produce cells that will populate the meristem, whereas the distal ICs produce cells of the columella (Dolan et al., 1993). The QC cells divide at a much lower rate than the ICs, although the frequency of division increases with the age of the plant (Timilsina et al., 2019). Clonal and time-lapse analyses have shown that QC divisions are asymmetric and replace different sets of ICs at different frequencies (Kidner et al., 2000; Wachsman et al., 2011; Cruz-Ramírez et al., 2013; Rahni and Birnbaum, 2019). Most QC cell divisions are periclinal (Figure 1B), producing two daughter cells that are positioned at different distances from the provascular cells of the root apical meristem (Cruz-Ramírez et al., 2013). The two daughter cells retain the activity of a QC marker for several days, until eventually one cell differentiates into a columella initial (Cruz-Ramírez et al., 2013). This indicates that QC cell divisions are symmetrical and produce identical cells, and that a cell fate asymmetry takes place after the division event. In this scenario, signals from the niche microenvironment might be instructive for this cell fate decision making. For instance, the production of columella initials is an emergent outcome of a system-level mechanism that considers the feedback regulation between the gene regulatory network in each cell and constraints in the expression pattern and intercellular mobility of the transcription factor SHORT ROOT (SHR) (Box 1; García-Gómez et al., 2020). The QC cells can also produce other types of ICs (Kidner et al., 2000; Rahni and Birnbaum, 2019); for instance, anticlinal QC divisions produce cortex/endodermis initials (Figure 1B; Rahni and Birnbaum, 2019). The QC cells are considered a reserve of multipotent SCs that can actively divide and replace lost or damaged initials and meristematic cells (Heyman et al., 2014). Interestingly, the root SCN organization in two SC populations with differing proliferative activities and generative potential is common to SCN of plants and animals (Barlow, 1978; Barlow, 1997; Jiang and Feldman, 2005; Li and Clevers, 2010), suggesting that this could be a generic feature of SCN organization.

**Box 1. A system-level mechanism regulating the asymmetric division of QC cells.**

The QC cells are considered a reserve of multipotent SCs that can produce all cell types in the root (Heyman et al., 2014), yet most QC cell divisions produce columella initials (Cruz-Ramírez et al., 2013). In a recent study, a mathematical model of genetic regulation in the root SCN was used to understand the mechanism behind this biased production of columella (García-Gómez et al., 2020). A perturbation analysis of a genetic regulatory model was used to identify the regulators that can cause cell state transitions *in silico* (García-Gómez et al., 2020); this represents the transition from one cell type to another. SHR was identified as a regulator that causes the transition from the QC to the columella initials state and thus as a candidate regulator that could be behind the asymmetric division of the QC cell. The constraints in SHR expression pattern, intercellular mobility, and nuclear retention in the cells of the RAM were studied in a multilevel model that recovered the dynamics reported upon QC cell divisions (Cruz-Ramírez et al., 2013), namely, that a periclinal QC cell division produces two QC cells, which over time develop differences in their intracellular levels of SHR due to their different proximity to the source of SHR. The intracellular SHR levels in each daughter cell are then interpreted by their regulatory networks, and for one daughter cell, this results in a transition to the columella initials state, resulting in asymmetry in cell fate (García-Gómez et al., 2020). The model also predicted that an increase in the availability of SHR causes a shift from asymmetric to symmetric QC cell divisions, increasing the pool of undifferentiated QC cells in the root SCN (García-Gómez et al., 2020).

The frequency of QC cell divisions changes with the developmental age of the seedlings in *Arabidopsis* and other plant species (Baum et al., 2002; Jiang and Feldman, 2005; Chen et al., 2011; Timilsina et al., 2019) and also shows variation in different *Arabidopsis* accessions (Aceves-García et al., 2016). Additionally, QC divisions can be stimulated in response to the availability of nutrients (Sánchez-Calderón et al., 2005), upon root meristematic damage and by genotoxic treatments (Cruz-Ramírez et al., 2013; Heyman et al., 2013). Plant hormones can be regulated by developmental and environmental cues at different levels, including metabolism, signaling, crosstalk, and transport, offering potential mechanisms to integrate external information into the regulation of SCN activity. The role of hormones as mediators between these cues and the regulation of SC activity in the root SCN is likely to be linked to the gene regulatory network that underlies QC identity and activity. In this case, plant hormonal responses could be channeled toward a common regulatory module to regulate the division at the QC according to the requirements of the plant.

In this review, we summarize current evidence regarding the regulation of QC cell division in the root SCN of *Arabidopsis*, focusing on how hormones interact with transcriptional regulatory networks implied in QC activity. We recapitulate on the transcription factors that have been identified as important regulators of QC specification; we summarize the information about the mitotic activity of the QC cells under optimal growth conditions, the role of reactive oxygen species (ROS), and several cell cycle components in the quiescence of the QC cells. We then discuss the effects of auxin, cytokinin (CK), brassinosteroids (BRs), and abscisic acid (ABA) on the division of the QC cells and on the expression of cell identity regulators. The existence of recurrent regulatory targets led us to discuss how hormonal responses may be channeled toward the genetic–hormonal regulatory network that underlies the acquisition of the cell identity and proliferative profiles in the root SCN, and how it can possibly constitute a developmental module to regulate SC activity in response to changing environmental conditions.

## Genetic Regulators of QC Cell Identity in the Root SCN

**FIGURE 2:**
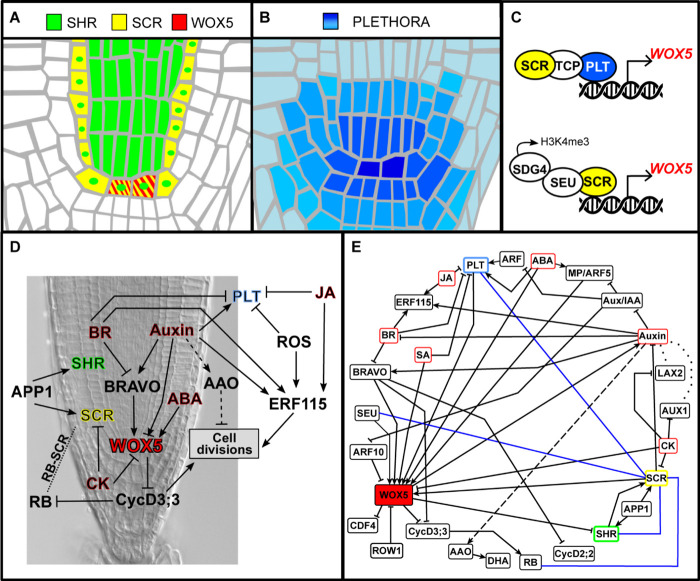
Expression and activity domains of the main genetic regulators of the QC cells fate. In **(A)**, SHR, SCR, and WOX5, and in **(B)**, the PLT family of transcription factors. **(C)** Protein complexes that bind to the regulatory regions of *WOX5* promoter. **(D)** Schematic representation of some of the regulatory interactions underlying the division of the QC cells. **(E)** Network depicting the regulatory interactions underlying QC divisions discussed throughout this review. The blue lines indicate protein–protein interactions; dotted lines indicate the role of auxin influx transporters, and the dashed line between auxin and AAO indicates that this particular regulation was observed in maize roots.

Several transcription factors have been identified as important regulators of QC cell identity; these also play important roles in the establishment of the radial pattern of the root and in the maintenance of the RAM. One of these regulators is the GRAS transcription factor *SHR* that is expressed in the provascular tissues at the RAM (Figure 2A; Benfey et al., 1993; Scheres et al., 1995; Helariutta et al., 2000). SHR moves to the endodermis, the cortex/endodermis ICs, and the QC (Nakajima et al., 2001), where it induces *SCARECROW* (*SCR*) expression (Cui et al., 2007). SCR and SHR form a heterodimer that localizes in the cell nucleus and restricts SHR’s intercellular movement (Cui et al., 2007). The SCR/SHR protein complex regulates the expression of genes necessary for the specification of the endodermis, the cortex/endodermis ICs, and the QC cells (Sabatini et al., 2003; Sarkar et al., 2007; Welch et al., 2007; Moreno-Risueno et al., 2015). Additionally, SHR forms heterodimers with JACKDAW (JKD), MAGPIE (MGP), and BLUEJAY (BLJ) transcription factors, forming different protein complexes that localize in the cell nucleus (Long et al., 2015; Long et al., 2017). It has been shown that the endodermis, cortex/endodermis initials, and the QC cells are enriched in different protein complexes containing SHR, SCR, and JKD (Long et al., 2017; Clark et al., 2020), which could be providing specificity in the genes that are regulated by SHR in the different cells of the adjacent layer to the provasculature (Long et al., 2015, 2017; Moreno-Risueno et al., 2015). Loss-of-function mutants in *scr* and *shr* have defects in the asymmetric division of the cortex/endodermis ICs, and the roots display a single layer of ground tissue (Di Laurenzio et al., 1996). Moreover, these mutants have defects in the specification of the QC and the ICs differentiate, leading to premature consumption of the meristem (Benfey et al., 1993; Sabatini et al., 2003; Sarkar et al., 2007).

The AP2-type PLETHORA transcription factors (PLT1, PLT2, PLT3, and PLT4/BABY BOOM [BBM]) are important regulators for root meristem maintenance (Aida et al., 2004; Galinha et al., 2007; Mähönen et al., 2014). The *PLT* transcription factors are highly expressed in the cells of the root SCN, including the QC (Figure 2B), and their expression is positively regulated by the high auxin levels in these cells (Aida et al., 2004; Galinha et al., 2007). A protein gradient of PLTs is created along the RAM because of cell growth and proliferation occurring in the meristem, as well as their intercellular movement (Mähönen et al., 2014). In this way, high PLT levels maintain the root SCN, intermediate levels maintain cell proliferation in the meristem, and low levels correlate with the beginning of cell differentiation (Mähönen et al., 2014). In the double-mutant *plt1 plt2*, the QC cells show defects in the expression of specific QC makers and display QC division events, indicating a loss of QC cell identity and its characteristic quiescent state (Aida et al., 2004).

Another important regulator of the root SCN is *WUSCHEL-related homeobox 5* (*WOX5*), a homeodomain transcription factor that is specifically expressed in the QC cells (Figure 2A; Sarkar et al., 2007). Mutant plants with non-functional WOX5 lack the expression of several QC-specific markers and display differentiation of the distal ICs (Sarkar et al., 2007; Ding and Friml, 2010). The expression of *WOX5* depends on the activity of the radial regulators SCR and SHR and the longitudinal PLT regulators mentioned in the previous paragraphs. The *scr* and *shr* mutants lack *WOX5* expression and display severe root growth defects (Sarkar et al., 2007), whereas in the case of *plt*, a multiple mutant has an expanded expression of *WOX5* compared to WT plants (Sarkar et al., 2007; Zhai et al., 2020). Recently, it was shown that PLT and SCR form a protein complex with teosinte-branched cycloidea PCNA (TCP) transcription factor to directly regulate *WOX5* expression and the identity of the QC cells (Shimotohno et al., 2018), thus showing a mechanism for the convergence of these regulatory pathways (Figure 2C). Moreover, SCR forms a transcriptional complex with SEUSS (SEU) at the promoter of *WOX5* (Figure 2C), in which SEU acts as a scaffold protein that recruits SET DOMAIN GROUP 4 (SDG4), a SET domain methyltransferase (Zhai et al., 2020). The transcriptional complex SCR-SEU-SDG4 is implicated in the deposition of the H3K4me3 epigenetic mark in the promoter of *WOX5*, critical for its expression and for QC specification (Zhai et al., 2020). *SCR* expression itself seems to be reduced in *seu* mutants, suggesting the existence of a positive feedback loop in the regulation of *WOX5* in the QC (Zhai et al., 2020).

Auxin is an important regulator of *WOX5*, and alterations in its distribution, for example, by altering its polar transport, result in the expression of *WOX5* in the endodermal cells of the meristem (Sabatini et al., 1999; Mähönen et al., 2014). Auxin signaling regulates *WOX5* positively and negatively through different auxin response factors (ARFs): MP (ARF5) is necessary for its expression, whereas ARF10/16 represses it (Sarkar et al., 2007; Ding and Friml, 2010). Interestingly, *MP* and *ARF10* are not expressed homogenously in the RAM (Rademacher et al., 2011), suggesting that the cellular context could be important to define the effect of auxin over *WOX5* (García-Gómez et al., 2017). Particularly, *MP* is expressed in the QC cells but not *ARF10* (Rademacher et al., 2011; Truskina et al., 2020), which raises the hypothesis that particular ARF profiles could be important for the auxin regulation of *WOX5* expression in these cells. Hence, it is of interest to uncover the mechanisms behind the expression patterns of these ARFs in the RAM in order to understand the specificity of auxin responses in the root meristem; however, there is still no evidence about it. Regulatory links between RAM patterning mechanisms and the auxin signaling components were postulated and put to the test through a mathematical model (García-Gómez et al., 2017). The hypothetical interactions analyzed with the model imply that the heterodimers formed between SHR and its interaction partners JKD and MGP might be involved in the regulation of *MP* and *ARF10*, namely, that the SHR-JKD heterodimer represses the expression of *ARF10*, whereas the SHR-MGP heterodimer represses the expression of *MP* (García-Gómez et al., 2017). These hypotheses are based on the bioinformatics prediction that SHR represses *ARF10* and *MP* expression (Levesque et al., 2006), on the binding of JKD to the promoter of *ARF10* (Moreno-Risueno et al., 2015), and the fact that *ARF10* and *JKD* are expressed on non-overlapping domains in the RAM, and the same for *MP* and *MGP* (Welch et al., 2007; Rademacher et al., 2011). The study of these hypothetical interactions in the context of a mathematical regulatory network model of the RAM showed that they are necessary to recover attractors (steady states) with the expression patterns of *MP* and *ARF10*, as observed in the cells of the root meristem, including the QC cells (García-Gómez et al., 2017). In the model, the recovered activity configurations allow the expression of *WOX5* in the QC cells, but not in the other RAM cells. The results from the model strongly suggest that the expression patterns of the ARF transcription factors define the effect of auxin over *WOX5*: cells of the RAM with different ARF10 and MP expression profiles will exhibit different auxin responses; some may activate *WOX5*, whereas other will repress it. The proposed links between patterning mechanisms and hormonal signaling pathways may be critical for understanding how cells will respond to auxin and may constitute a generic mechanism for the spatial specificity of hormonal responses in plant development. Interestingly, it has been shown that the chromatin of several ARFs is constitutively open for transcription, and a series of transcriptional repressors affect their expression (Truskina et al., 2020). Under such a scenario, SHR-JKD and SHR-MGP could act as the repressors that are behind the expression patterns of *ARF10* and *MP*, respectively, and that underlie the spatial specificity of their activity in the RAM.

Another important regulator of *WOX5* is REPRESSOR OF WUSCHEL1 (ROW1), a PHD domain-containing protein that has been shown to restrict *WOX5* expression to its characteristic position at the center of the root SCN (Zhang et al., 2015). WOX5 activity in the QC cells is important for the maintenance of the ICs (van den Berg et al., 1997; Ding and Friml, 2010), and in the case of the distal ICs, this is achieved, in part, via the non-cellular autonomous activity of WOX5 (Pi et al., 2015). WOX5 moves from the QC cells to the distal ICs where it recruits corepressors and a histone deacetylase to repress the expression of *CYCLING DOF FACTOR 4* (*CDF4*), which promotes the terminal differentiation of the columella cells (Pi et al., 2015). WOX5 also moves toward the provascular initials, where it has been proposed to negatively regulate the expression of *SHR* (Clark et al., 2020).

## Cell Cycle Regulation of the QC Cells

The QC cells divide in optimal growth conditions (Timilsina et al., 2019), albeit at a lower frequency compared to the surrounding ICs and meristematic cells (Wachsman et al., 2011; Cruz-Ramírez et al., 2013). In a pioneering article by Clowes (1956), it was estimated that the QC cells in maize roots display a quarter of DNA synthesis compared to meristematic cells (Clowes, 1956), which is in remarkable accordance to what has been reported for *Arabidopsis* (Cruz-Ramírez et al., 2013). This similarity in the division frequencies suggests the existence of generic patterns of cell cycle regulation in the root meristem of different plant species.

In *Arabidopsis*, root growth is not compromised by genetic perturbations that result in alterations in the division patterns of the QC cells, suggesting that low division rate of the QC cells is not strictly necessary for the function and the organization of the root meristem under optimal growth conditions (Vanstraelen et al., 2009; González-García et al., 2011; Cruz-Ramírez et al., 2013; Savina et al., 2020; Wang et al., 2020). The division of the QC cells has been shown to increase in frequency in older *Arabidopsis* seedlings (Timilsina et al., 2019), and it can also be actively modulated to cope with the current needs of the root. For instance, as a response to changes in hormonal activity, limiting phosphate conditions (Sánchez-Calderón et al., 2005), genotoxic treatments that cause cell death of the proliferating cells of the meristem (Cruz-Ramírez et al., 2013; Heyman et al., 2013; Vilarrasa-Blasi et al., 2014), or after the excision of the root cap (Ponce et al., 2005). In the case of QC cell divisions that occur as a response to meristematic damage, it is unknown what non–cell-autonomously mechanism is involved in the modulation of QC cell divisions. A potential mechanism to achieve this could be the directional signaling from mature cells of the meristem to the SCs to maintain cell fate (van den Berg et al., 1995), although this possibility requires further investigation.

The regulation of QC cell divisions, as a response to endogenous or environmental signals, must ultimately impact on the activity of the regulators that underlie the progression of the different phases of the cell cycle (Polyn et al., 2015; Ortiz-Gutiérrez et al., 2015). Interestingly, the transcription factor WOX5 directly inhibits the expression of *CYD3;3* (Forzani et al., 2014), implying a direct regulatory link between a QC cell fate transcription factor and a regulator of the progression of the cell cycle. D-type cyclins (CYCD) form a complex with A-type cyclin-dependent kinases to regulate the commitment point at G1/S transition through the phosphorylation and inactivation of retinoblastoma-related (RBR) protein, to release E2F transcriptional factor (Polyn et al., 2015); these are necessary steps for the transition to the S phase of the cell cycle. Otherwise, RBR activity maintains a quiescent state of the QC cells (Wildwater et al., 2005; Cruz-Ramírez et al., 2013). RBR in the QC maintains its low proliferative state, and consequently seedlings with no RBR activity in the QC display cell divisions (Cruz-Ramírez et al., 2013). This regulation of QC cell proliferation is mediated by the interaction of RBR and SCR (Cruz-Ramírez et al., 2013). Disruption of this interaction yields QC divisions (Cruz-Ramírez et al., 2013), thus establishing a regulatory circuit of cell cycle and cell fate regulators that modulate QC cell divisions. The repression of *CYCD3;3* by WOX5 explains the extended G1 phase and low mitotic rate of the QC cells. Interestingly, local expression of *CYCD1;1* and of *CYCD3;3* in the QC using the *WOX5* promoter showed that only CYCD3;3 is able to significantly induce cell division in the embryonic QC (Forzani et al., 2014). Additionally, *CYCD6;1*, which is part of a regulatory circuit that regulates the asymmetric cell division of the cortex/endodermis IC, is not expressed in the QC cells (Sozzani et al., 2010; Cruz-Ramírez et al., 2013). Hence, CYCD proteins might be part of cell type–specific programs of cell cycle regulation, which could underlie the varying proliferation rates in different tissues (de Almeida Engler et al., 2009).

Protein degradation processes regulate the progression of the cell cycle (Gutierrez, 2009), and they have been found important for the control of QC cell divisions (Ueda et al., 2004; Vanstraelen et al., 2009). For example, *HALTED ROOT* (*HLR*) encodes a subunit of the ubiquitin 26S proteasome, and the *hlr* mutant displays dividing QC cells and a loss of the expression of characteristic markers of these cells (Ueda et al., 2004). It has been shown that the *hlr* mutant is defective in auxin signaling, as the degradation of IAA17 is compromised (Ueda et al., 2004). IAA17 is a member of the family of AUX/IAA repressors (Box 2), which interact with the ARF transcription factors and impede them to regulate the expression of auxin-responsive genes (Ulmasov et al., 1997, 1999; Okushima et al., 2005; Guilfoyle and Hagen, 2007). Notably, IAA17 and WOX5 act in the same regulatory pathway in the QC cells (Ding and Friml, 2010; Tian et al., 2014). An IAA17 gain-of-function mutant has altered auxin levels in the QC cells, multiple layers of the distal ICs, and an expanded *WOX5* expression domain (Ding and Friml, 2010; Tian et al., 2014). This phenotype clearly shows that if the degradation of IAA17 is compromised, as observed in the *hlr* mutant, there will be defects in the organization of the QC cells and the SCN (Ueda et al., 2004). The defects of the IAA17 gain-of-function mutant concerning the restriction in the expression of *WOX5* could be related to the regulation of MP and ARF10, as both interact with this particular AUX/IAA repressor (Vernoux et al., 2011; Piya et al., 2014) and both regulate *WOX5*, as it was mentioned in the previous section.

**Box 2. Hormone signal transduction basics.**

• Auxin

Auxin regulates a high variety of plant developmental processes, including cell proliferation in the root meristem and the maintenance of the root SCN (Sabatini et al., 1999; Ishida et al., 2010). The auxin signaling pathway is composed of the family of ARF and Aux/IAA transcription factors that regulate the expression of auxin-responsive genes (Ulmasov et al., 1999; Okushima et al., 2005; Guilfoyle and Hagen, 2007). The auxin signaling pathway is elicited when the hormone binds to its coreceptors, the transport inhibitor response1/auxin signaling F-box protein1-5 (TIR1/AFB) and its substrates, the Aux/IAA proteins (Dharmasiri et al., 2005; Calderon Villalobos et al., 2012). TIR1/AFB are components of the SKP1/Cullin/F-box protein (SCF^*TIR1/AFB*^) ubiquitin ligase complex, and auxin produces a conformational change that favors its interaction with the Aux/IAA proteins, promoting their ubiquitination and eventual degradation (Xu et al., 2007). In this way, auxin frees the ARF transcription factors from the repressive action of Aux/IAA, so that they can regulate the expression of auxin-responsive genes.

Auxin displays a concentration gradient along the longitudinal axis of the root with a maximum at the QC cells (Sabatini et al., 1999; Sarkar et al., 2007; Petersson et al., 2009; Brunoud et al., 2012). This gradient correlates with the cellular activities of the cells along the RAM: the highest auxin concentration is found in the SCN, where cells have low division rates; the proliferation domain has high auxin concentration, and cells divide actively, and then in the transition domain, where auxin levels decrease, and cells stop dividing (Blilou et al., 2005; Mähönen et al., 2014). Auxin distribution in the root is the result of the regulation of auxin metabolism, conjugation, and transport, the latter mediated by efflux and influx proteins that actively move auxin between cells (Petersson et al., 2009; Vanneste and Friml, 2009; Liu et al., 2017). Auxin can enter cells passively and also through the activity of the auxin influx proteins AUX1, LAX1, LAX2, and LAX3, which are expressed in different tissues of the root (Swarup et al., 2005; Péret et al., 2012). The family of PIN-FORMED (PIN) proteins are auxin efflux transporters that play a major role in the generation of auxin distribution patterns throughout development (Blilou et al., 2005). In the root, the PIN efflux transporters are polarly localized in the cell membranes, forming a rootward auxin flux through the vascular tissues. At the columella, PINs redistribute auxin laterally, connecting it with a shootward flux through the outside root tissues (Blilou et al., 2005). This PIN distribution forms a transport network that underlies the distribution of auxin in a gradient with a maximum in the position of the QC cells (Blilou et al., 2005; Grieneisen et al., 2007). The distribution of auxin in the root meristem is tightly regulated and can be modulated by complex mechanisms that regulate *PIN* expression and PIN localization and the regulation of auxin metabolism that fine-tunes the patterns of auxin accumulation in the cells (Gonzali et al., 2005; Liu et al., 2017).

• Cytokinin

Plant cells sense CK via a two-component signaling pathway similar to the phosphorelay system found in bacteria (Santner et al., 2009). The CK receptors, *Arabidopsis* His kinase 2 (AHK2), AHK3 and cytokinin response 1 (CRE1)/AHK4, are transmembrane proteins that autophosphorylate upon CK binding and transfer the phosphoryl group to *Arabidopsis* His-phosphotransfer proteins (AHP). Eventually, AHP proteins translocate to the nucleus and the signal is transferred to the Arabidopsis response regulators (ARRs) transcription factors. There are four types of ARR proteins based on their protein similarity (To et al., 2007). Type B ARRs positively regulate the expression of CK responsive genes, including the type A ARRs that repress CK signaling. Additionally, the type B ARRs promote the expression of the cytokinin response factor (CRF) family of transcription factors (Rashotte et al., 2006). The CRF proteins accumulate in the nucleus depending on the activity of AHP proteins to regulate the expression of CK-responsive genes (Rashotte et al., 2006). The type C ARRs have phosphatase activity and are thought to regulate CK signaling negatively by removing the phosphoryl group from type B and type A ARRs (Kiba et al., 2004). The fourth group corresponds to the *Arabidopsis* pseudoresponse regulators that has been shown to participate in the regulation of the circadian rhythm (To et al., 2007).

• Brassinosteroids

The steroid hormones BRs are perceived in the plasma membrane by a group of leucine-rich repeat receptor-like kinase (LRR-RKL) receptors (brassinosteroid insensitive 1, BRI, and brassinosteroid receptor-like 1 BRL1 and BRL3 in *Arabidopsis*) that, upon BR binding, elicit a signal transduction cascade that inhibits BR-insensitive 2 (BIN2) (Zhu et al., 2013). In the absence of BR, BIN2 phosphorylates the transcription factors EMS suppressor 1 (BES1) and brassinazole-resistant 1 (BZR1), blocking their ability to bind their DNA targets (Belkhadir et al., 2014). Upon BR binding by the receptors, a signaling cascade is induced that ultimately results in dephosphorylation and increased nuclear localization of BES1/BZR1, which can in turn regulate the expression of BR-responsive genes (Belkhadir et al., 2014).

• Abscisic Acid

The ABA signaling pathway is elicited when the hormone binds to the PYR/PYL/RCAR receptor proteins that release SnRK2s kinases from PP2Cs inhibition, thereby activating the ABF/AREB transcription factors to regulate the expression of ABA-responsive genes (Santner et al., 2009).

The CELL CYCLE SWITCH 52 A1 (CCS52A1) and CCS52A2 protein isoforms are components of the ANAPHASE-PROMOTING COMPLEX/CYCLOSOME (APC/C) that targets several cell cycle proteins for degradation, important for cell cycle progression and mitosis (Vanstraelen et al., 2009). Interestingly, APC regulates mitotic arrest in the QC and also the onset of endocycle in the transition domain of the root meristem (Vanstraelen et al., 2009; Heyman et al., 2013; Takahashi and Umeda, 2014). In the *ccs52a2* loss-of-function mutant, the QC cells divide more frequently than in wild-type plants, and the meristem is eventually exhausted (Vanstraelen et al., 2009; Heyman et al., 2013). One of the regulatory targets of CCS52A2 is ETHYLENE RESPONSE FACTOR 115 (ERF115), whose expression is observed prior to the division of QC cells (Heyman et al., 2013). ERF115 activates the expression of *phytosulfokine5* (*PSK5*), a peptide hormone that induces QC cell divisions (Heyman et al., 2013). Overexpression of *ERF115* results in a marked increase in the frequency of QC cell divisions in the root SCN, indicating that it is a positive regulator of QC mitotic activity. Although *ERF115* has been annotated as an ethylene response factor, its expression is actually not regulated by ethylene. Instead it is induced by ROS signaling (Kong et al., 2018) and brassinolide (BL) treatment (Heyman et al., 2013). Notably, QC cell divisions still take place in *erf115* mutants treated with BL (Heyman et al., 2013), indicating that BR also promotes cell divisions independently of ERF115 (Vilarrasa-Blasi et al., 2014).

Recently, it was found that ULTRAPETALA1 (ULT1), described as a trithorax (TrxG) component, is also required for QC cell divisions, evidencing the participation of the epigenetic factors in this process (Ornelas-Ayala et al., 2020).

## Redox Regulation of QC Cell Division

Redox regulation plays a critical role in the organization of the RAM in *Arabidopsis* (Tsukagoshi et al., 2010), but its role in the QC is not so clear. In this section, we include research studies from maize regarding the function of redox regulation in the QC cells, in order to provide insights of its role in *Arabidopsis*.

In maize roots, the boundary between the QC and the proliferating cells of the meristem is marked by a drastic change in the redox cellular state (Kerk and Feldman, 1995; Jiang et al., 2003). The position of the QC in the root apex is characterized by an oxidizing environment with high levels of dehydroascorbic acid (DHA) and glutathione disulfide, whereas a reduced state is detected in the neighboring cells in the RAM with high levels of ascorbic acid (AA) and glutathione (GSH) (Kerk and Feldman, 1995; Jiang et al., 2003). The redox profiles of the quiescent SCs and the proliferative meristematic cells could be important in the definition of these zones of contrasting mitotic activity. This notion is supported by experiments in which the QC cells start dividing in maize roots treated with AA, whereas cells become arrested in the G1 phase of the cell cycle when roots are treated with an inhibitor of AA biosynthesis (Kerk and Feldman, 1995, and references therein), indicating the importance of the redox status in the regulation of QC cell divisions.

Reduced compounds such as AA and GSH, which are enriched in meristematic cells of maize, are necessary for the progression of many generic cellular processes including the transition from G1 to S phase of the cell cycle, metabolic reactions, and protein synthesis (Kerk and Feldman, 1995; Vernoux et al., 2000; Jiang and Feldman, 2005; De Tullio et al., 2010). The molecular mechanism behind these effects may involve these molecular species acting as second messengers in signaling pathways (Apel and Heribert, 2004) and in the regulation of protein activity and conformation. In *Arabidopsis*, this could be mediated through the oxidation/reduction of cysteine residues in enzymes and transcription factors (De Tullio et al., 2010), which potentially could modulate the information processing capabilities of the cells. AA has been suggested to affect ethylene biosynthesis (Arrigoni and Tullio, 2000). As ethylene induces QC cell division (Ortega-Martínez et al., 2007), this potentially represents another mechanism by which the redox status of the cell regulates QC cell division.

In *Arabidopsis*, several reports indicate the importance of the redox status of the QC cells in the maintenance of their low mitotic rate. For instance, the *app1* mutant, a mutant in a mitochondrial ATPase, has altered levels of reactive oxygen species (ROS) in the cells of the RAM and displays an increase in QC cell division (Yu et al., 2016; Kong et al., 2018). Interestingly, this phenotype is accompanied by a reduction in the expression of the transcription factors *SCR* and *SHR* (Yu et al., 2016). Salicylic acid (SA) is a hormone that plays an important role in plant defense, and it induces QC cell divisions in a dose-dependent manner (Wang et al., 2020). The SA-induced cell divisions are mediated by an increase in ROS levels the RAM and a downregulation of *PLT1*, *PLT2*, and *WOX5* in the QC cells (Wang et al., 2020). It was also previously shown that increased ROS levels cause a downregulation of *PLT* genes, a higher expression of *ERF115*, among other factors (Kong et al., 2018). Altogether, these studies in *Arabidopsis* indicate a key role of redox regulation in QC cell divisions and show the existence of interesting links between QC cell identity and its proliferative state.

It is remarkable that in neural SCs, ROS production in mitochondria has also been shown to regulate SC fate by regulating the expression of key developmental genes (Khacho et al., 2016), suggesting that this could be a generic mechanism for the control SC activity as a response of the internal redox state of the cells.

## Hormonal Regulation of QC Mitotic Activity

Auxin and CK, as well as BR and ABA, have antagonistic roles in different developmental contexts, including the division of the QC cells in the root SCN. In this section, we review the regulatory crosstalk between these two pairs of antagonistic hormones. All interactions were included in a network that illustrates the complexity underlying QC cell division (Figure 2E). Other plant hormones such as gibberellins are not included in this review because it has been demonstrated to regulate root growth independently of the activity of the SCN (Achard et al., 2009; Ubeda-Tomás et al., 2009; González-García et al., 2011).

### Auxin and CK Antagonism in the Regulation of the QC Cell Divisions

In the root SCN, WOX5 promotes auxin accumulation in the QC by inducing the expression of the auxin biosynthetic enzymes *YUCCA1* (Tian et al., 2014), *tryptophan aminotransferase of arabidopsis1* (TAA1; Savina et al., 2020), and by repressing the expression of auxin conjugation genes (Gonzali et al., 2005). As *WOX5* expression is induced by auxin in the QC (Sarkar et al., 2007), this establishes an auxin—WOX5-positive feedback loop in these cells. Moreover, SCR controls auxin levels in the QC cells by indirectly repressing the expression of *ASB1* (*ANTHRANILATE SYNTHASE BETA SUBUNIT 1*), an enzyme involved in auxin biosynthesis (Moubayidin et al., 2013). Consequently, in the *scr-1* mutant, the auxin content is dramatically increased, and the SCN is disorganized (Moubayidin et al., 2013). This suggests that auxin levels have to be actively modulated in the QC cells, to maintain appropriate levels for the long-term organization of the root SCN.

In the QC cells, auxin indirectly promotes low division rates through the positive regulation of *WOX5*, maintaining low levels of *CYCD3;3* in these cells (Figure 2D). A study from maize suggests another mechanism by which auxin may impact on QC cell divisions. In maize roots, auxin promotes the expression and the activity of the enzyme ascorbate oxidase (*AAO*), which oxidizes AA to DHA (Kerk and Feldman, 1995). *AAO* expression is high in the QC, moderate in the meristem, and absent in the mature root (Kerk and Feldman, 1995), correlating with the auxin concentration gradient along the RAM. As mentioned in the previous section, in maize, the QC cells have a redox status different to that of the meristem cells. The spatial distribution of auxin and *AAO* along the RAM suggests that the redox status of the cells may be established, at least in part, by auxin. In support of this idea, maize roots treated with 1-*N*-naphtylphthalamic acid (NPA), an inhibitor of auxin efflux transport, display changes in auxin distribution, and the QC becomes less oxidized (Jiang et al., 2003). This change in the redox state of the QC preceded the incorporation of the nucleotide analog, BrdU, strongly suggesting that this change in the redox status of the QC cells underlies the increase in their proliferation rate (Jiang et al., 2003). Based on these results, it was proposed that high levels of auxin in the QC cells regulate the redox status of the cells and maintain low proliferation rates of the QC cells (Jiang et al., 2003). It remains to be determined if this redox regulation also occurs in *Arabidopsis*. Experiments in *Arabidopsis* indicate that SA-induced QC cell divisions are accompanied by an increase in ROS levels and a decrease in auxin signaling in the QC cells (Wang et al., 2020), thus suggesting the existence of a mechanism similar to the one described in maize roots.

Cytokinins have an antagonistic function to auxin in different developmental processes. For instance, the crosstalk between these hormones regulates the balance between proliferation and differentiation in the RAM (Moubayidin et al., 2009; Su et al., 2011; Aichinger et al., 2012; Rodriguez-Villalon and Hardtke, 2014). In the QC, these hormones also have antagonistic role as CK induces cell division. Plants with increased CK signaling display ectopic division of the QC cells (Zhang et al., 2011, 2013). For example, the *arr3,4,5,6,7,8,9,15* loss-of-function multiple mutant in numerous type A ARRs results in CK hypersensitivity and a higher rate of cell division in the QC compared with wild-type plants (Zhang et al., 2011). This phenotype is accompanied by the differentiation of the distal ICs and mild alterations in the auxin response of the QC, indicating that type A ARRs are necessary for maintaining appropriate activity of the QC (Zhang et al., 2011).

Wild-type roots treated with exogenous CK and CK oxidase mutants (*ckx3* and *ckx5*) with elevated endogenous levels of CK also show an increase in QC cell division (Zhang et al., 2013). Expression analyses showed that *WOX5* and *SCR*, as well as the auxin influx transporters *AUX1* and *LAX2*, are down-regulated in the QC of these mutants (Zhang et al., 2013). Interestingly, ARR1 (type B ARR) directly binds to the promoter of *LAX2*, which is expressed in the provascular tissues and the QC cells (Péret et al., 2012), and the QC cells divide in the *lax2* mutant (Zhang et al., 2013). Furthermore, in roots treated with exogenous CK, *LAX2* expression is repressed, resulting in dampening auxin accumulation in the QC cells. Hence, the induction of QC cell division by CK could be an indirect result of lowering auxin levels in the QC. This evidence agrees with a notion where high auxin concentration in the QC promotes a state of no cell divisions. It is interesting that CK levels in the QC cells are rather low, whereas these increase in the neighboring cells (Zhang et al., 2013; Zürcher et al., 2013; Antoniadi et al., 2015), suggesting that a tight spatial regulation of CK metabolism and signaling is important to maintain the QC cells.

### Effects of BRs and ABA in the Regulation of QC Cell Divisions

Treating wild-type seedlings with exogenous L-brassinolide (BL) induces the division of the QC cells and the differentiation of distal ICs in a dose-dependent manner (González-García et al., 2011; Fàbregas et al., 2013). Accordingly, the gain-of-function *bzr1-1d* has actively dividing QC cells even if BR biosynthesis is blocked (Chaiwanon and Wang, 2015). Two of the three known BR receptors, namely, BRL1 and BRL3, are detected mainly in the root SCN (Fàbregas et al., 2013), and the*ir* loss-of-function mutants show a reduction in the division rate of the QC cells in comparison with wild-type seedlings (Fàbregas et al., 2013). The protein of the third BR receptor, BRI1, is detected in the root meristem but not in the QC (van Esse et al., 2011; Fàbregas et al., 2013). Interestingly, despite this apparent absence of BRI1 in the QC cells, it is necessary for BR-induced QC cell divisions, as in the *bri1-116* mutant the divisions were completely abolished in roots treated with BL (Vilarrasa-Blasi et al., 2014).

The nuclear accumulation of BES1 and BZR1 can be used as a marker of the activity of the BR signaling. In the SCN, these proteins accumulate mostly in the cytoplasm indicating that the BR signaling pathway is not active in these cells (Chaiwanon and Wang, 2015). As the QC cells of young *Arabidopsis* roots are mitotically quiescent, endogenous mechanisms to maintain BR signaling low in these cells may exist. Based on current evidence, this might be mediated by a low accumulation of BRI1 protein in the QC cells (van Esse et al., 2011; Fàbregas et al., 2013) and by the auxin-dependent increased local BR catabolism in the root SCN area (Chaiwanon and Wang, 2015).

Brassinosteroids signaling negatively affects the expression of a significant number of QC-enriched genes, suggesting that loss of QC identity is linked to an increase in its proliferation (Chaiwanon and Wang, 2015). The MYB transcription factor BRASSINOSTEROIDS AT VASCULAR AND ORGANIZING CENTER (BRAVO) was identified as the only BR-regulated gene that is a direct target of BES1 and BZR1 in the proximal ICs and the QC cells (Vilarrasa-Blasi et al., 2014). *BRAVO* expression is reduced upon BL treatment in a time- and dose-dependent manner, and this reduction occurs before the BL-induced QC cell divisions (Vilarrasa-Blasi et al., 2014). The *bravo* loss-of-function mutant has increased mitotic activity of the QC cells and a dramatic reduction in the expression of *WOX5* and other QC markers (Vilarrasa-Blasi et al., 2014). In the roots of ectopic expression inducible lines of *BRAVO*, several cell cycle genes are downregulated including *CYCD2;2* and *CYCD3;3*, providing clues of the mechanism by which BRAVO may impact on the cell cycle progression to repress QC divisions. Intriguingly, loss-of-function *wox5-1* mutants show resistance to BR with respect to QC cell proliferation (González-García et al., 2011), indicating that WOX5 is a crucial regulator for the BR-induced QC divisions. This evidence supports a conceptual model where QC cell identity is intimately linked with cell division. In the case of BR, the activation of QC cell divisions may be mediated, in part, by relieving the WOX5-dependent inhibition of *CYCD3;3* (Figure 2D). As both BRAVO and WOX5 regulate negatively the expression of *CYCD3;3* (Forzani et al., 2014; Vilarrasa-Blasi et al., 2014), it is tempting to speculate that BRAVO acts through WOX5 in the regulation of QC mitotic activity. Additionally, as BRAVO affects the expression of other cell cycle regulators, it is likely that it also regulates QC cellular quiescence through a parallel pathway (Vilarrasa-Blasi et al., 2014).

On the other hand, ABA has been reported to maintain the quiescent state of the QC cells. Indeed, the low division rate of the QC cells is compromised in ABA-deficient and ABA-insensitive mutants, and in wild-type plants treated with fluridone, an inhibitor of ABA biosynthesis (Zhang et al., 2010). Roots of these plants display increased differentiation of distal ICs, and in some cases, the QC cells had starch granules (Zhang et al., 2010), suggesting that the function and identity of the QC are severely compromised. On the contrary, exogenous ABA treatment induced the quiescence of the QC cells, reduced distal IC differentiation, and increased the expression of root SCN regulators, as *PLT2*, *MP*, and *WOX5* (Zhang et al., 2010). WOX5 mediates the effect of ABA in preventing distal IC differentiation, as treatment of *wox5-1* mutants with either ABA or fluridone no longer altered the differentiation pattern of distal IC (Zhang et al., 2010). Moreover, it has been reported that overexpression of *WOX5* (*35S:WOX5*) potentiates ABA effects related to the additional distal ICs files (Sarkar et al., 2007; Zhang et al., 2010). Surprisingly, distal ICs became differentiated when *35S:WOX5* plants were treated with fluridone, strongly suggesting that the effect of WOX5 over distal ICs depends on ABA availability (Zhang et al., 2010). Altogether, the mentioned evidence indicates that ABA promotes the quiescence of the QC cells, in part by promoting the expression of *WOX5* among other transcription factors, and there might exist a mutual interdependency between ABA and WOX5 to regulate the differentiation of distal ICs.

In summary, the antagonistic effects of BR and ABA on QC cell division are mediated in part by the regulation of QC cell factors, including WOX5. Interestingly, ABA treatment causes a slight increase of *BRAVO* expression (Vilarrasa-Blasi et al., 2014), suggesting that it could be a mediator of BR and ABA responses in the QC cells, although it remains to be determined if this is indeed the case.

## An Integrative Regulatory Module for QC Cell Identity and Cell Divisions

The regulatory interactions related to the division of the QC cells described in the previous sections were integrated in a regulatory network that might constitute a developmental module of SC regulation (Figure 2E). Through this conceptual framework, it is possible to get insight into how each hormone is affecting the activity of the other elements of the network, and then understand how the system overall is responding to hormonal alterations. For instance, it can be noticed that WOX5 is a recurrent target in the hormonal regulation of QC cell division, making it a central component of the proposed regulatory module (Figure 2E). This convergent regulation of a QC-specific transcription factor suggests that the regulation of QC cell division by hormonal signaling pathways is intimately linked with QC cell identity (Figure 2). In this regard, it is remarkable that WOX5 directly represses *CYCD3;3* (Forzani et al., 2014) because this establishes a direct link between cell fate regulation in the QC and mitotic quiescence. However, the low proliferation state of the QC cells might be maintained by other means, for example, by the activity of the proteasome (Ueda et al., 2004; Vanstraelen et al., 2009), through the direct regulation of various cell cycle components (Vilarrasa-Blasi et al., 2014), or, as suggested by studies from maize, by the regulation of the redox status of the cells (Kerk and Feldman, 1995; Jiang et al., 2003).

The notion that hormones are channeled toward a common regulatory module to adjust QC cell divisions is supported by reported antagonistic effects on the regulation of QC genes and of QC cell division. For example, auxin and BR have antagonistic effects in the regulation of QC quiescence, and most of the genes that are repressed by BR in the QC are induced by auxin (Chaiwanon and Wang, 2015). *BRAVO* and all *PLTs* are part of the genes regulated differentially by both auxin and BR (Chaiwanon and Wang, 2015), thus establishing a mechanism by which both hormones impact on the cell fate of the QC cells (Figures 2D,E). Auxin also promotes the expression of BR catabolic enzymes in order to maintain BR low levels in the root meristem cells and establishing auxin and BR domains with no overlapping responses (Chaiwanon and Wang, 2015). Thus, there are many ways in which this hormonal crosstalk takes place, and its study can be aided through a network approach.

In the case of auxin and CK, this pair of hormones has antagonistic roles in the regulation of cell division in both the RAM and in the QC cells. Intriguingly, auxin promotes cell proliferation in the meristem and mitotic quiescence in the QC (Kerk and Feldman, 1995; Ishida et al., 2010; Chaiwanon and Wang, 2015), whereas CK promotes the opposite (Dello ioio et al., 2008; Zhang et al., 2013). This indicates that there is a general antagonism between auxin and CK that is independent of the tissue context (Table 1). The regulatory crosstalk between auxin and CK is not necessarily conserved in the root meristem and the QC (reviewed in Garay-Arroyo et al., 2012; Table 1). For example, CK induces the expression of the Aux/IAA repressor *SHY2* to regulate meristem size, and this is a key point in the regulatory crosstalk between auxin and CK (Dello ioio et al., 2008), but SHY2 is not involved in the regulation of CK-induced QC cell division (Zhang et al., 2013). Therefore, how the hormonal regulatory modules act in the meristematic cells and in the QC, and how they are coupled remain to be uncovered. The opposite effects of these hormones in the meristem and the QC could be due to quantitative variations in its levels and the specific gene activity profile in each context (Fridman et al., 2014; Vragović et al., 2015). Thus, considering the gene regulatory network that underlies the acquisition of different fates in the RAM could be very instrumental to understand the opposite effects of these hormones in the different zones of the root apex (García-Gómez et al., 2017).

**TABLE 1:** Hormonal regulatory effects in the QC and root meristem cells.

Hormone	Link to primary metabolism	Effect in the QC	Effect in the meristem	Regulation of SCN transcription factors	Other regulated genes at the root tip	References
Brassinosteroids	—	Proliferation	Proliferation and differentiation	WOX5, PLT1, PLT2, BBM, and AGL42.	BRAVO and KRP2	González-García et al., 2011; Vilarrasa-Blasi et al., 2014; Vragović et al., 2015; Chaiwanon and Wang, 2015
Abscisic acid	—	Quiescence	Proliferation and differentiation	WOX5, MP, and PLT2		Zhang et al., 2010
Auxin	Tryptophane	Quiescence	Proliferation	WOX5, PLT1, PLT2, BBM, and BRAVO	AAO (in maize roots) and BR catabolic enzymes	Kerk and Feldman, 1995; Aida et al., 2004; Galinha et al., 2007; Sarkar et al., 2007; Chaiwanon and Wang, 2015
Cytokinin	Adenine	Proliferation	Differentiation	WOX5 and SCR	LAX2, SHY2 and CCS52A1	Dello ioio et al., 2008; Takahashi and Umeda, 2014; Zhang et al., 2013
Jasmonic acid	Isoleucine	Proliferation	Differentiation	PLT1 and PLT2		Chen et al., 2011
Ethylene	Methionine	Proliferation	—	—	TAA1	Stepanova et al., 2008

It is interesting that there are mutants with RAM defects, which display QC cell divisions despite maintaining *WOX5* expression, indicating that, although it is a central component in the hormonal regulation of QC cell division, it is not enough to maintain a quiescence cell state. Examples of this are the *ccs52a2* loss-of-function mutant (Vanstraelen et al., 2009), BR gain-of-function signaling mutants (González-García et al., 2011), a mutant with SA overaccumulation (Wang et al., 2020), a down-regulation of *rbr* in the QC cells (Cruz-Ramírez et al., 2013), and mutants affecting folate metabolism (Reyes-Hernández et al., 2014) and threonine synthesis (Reyes-Hernández et al., 2019). As reviewed here, other important regulators of QC divisions include the redox status of the cells (Kerk and Feldman, 1995; Jiang et al., 2003) and BRAVO, which controls the expression of several cell cycle genes (Vilarrasa-Blasi et al., 2014). This could constitute parallel ways in which the division of the QC cells can be modulated independently of WOX5. As we learn more about the effects of hormones on the activity of the cell fate regulators of the root SCN, these could be integrated into the network to assess their effect on the other key elements of QC cell regulation (Figure 2E).

Regarding jasmonic acid (JA), a report showed that it induces QC cell proliferation, and it has been suggested to be through the control of the transition from the G2 to M phase of the cell cycle (Chen et al., 2011). Additionally, JA signaling inhibits the expression of the auxin-responsive genes *PLT1* and *PLT2* (Chen et al., 2011), and recent reports show that it promotes QC cell division through the RBR-SCR regulatory circuit and ERF115 (Zhou et al., 2019), thus connecting JA signaling with the regulatory module controlling cell fate and division in the QC. Moreover, auxin induces the expression of *ERF115* during regeneration as QC cell divisions take place (Zhou et al., 2019), indicating a multistability of auxin signaling in the regulation of QC cell divisions. Ethylene also promotes the proliferation of the QC cells, but the molecular mechanism is currently unknown (Ortega-Martínez et al., 2007). It has been reported that this ethylene effect on QC cell division is achieved independently of auxin, BR, CK, and JA (Chen et al., 2011; Heyman et al., 2013; Zhang et al., 2013). Curiously, in maize roots, NPA-induced QC cell divisions are reverted by cotreatment with an ethylene precursor ACC, indicating a regulatory interaction between these hormones in the regulation of the QC (Ponce et al., 2005). In this study, it is suggested that this might be a non–cell-autonomous effect mediated by a deregulation of auxin transport (Ponce et al., 2005).

Finally, the hormonal regulatory interactions that underlie QC cellular quiescence are non-linear and occur in a multicellular context, so an integrative approach of regulatory networks could aid in understanding these interactions (Azpeitia and Alvarez-Buylla, 2012; García-Gómez et al., 2020).

## Perspectives

The interconnection of hormonal signaling pathways, regulators of cell division, and the cell identity of the QC cells is an exciting matter of research that could reveal systemic mechanisms by which SC activity in plants is dynamically modulated to adapt to changing environmental and physiological conditions. Although this is of interest to the field of plant development, recent reports in animal SCNs are finding features that are also present in plant SCNs (Li and Clevers, 2010), and thus, what we learn about the QC regulation could potentially uncover generic regulatory mechanisms of SCs. Some of the most remarkable similarities between plant and animal SCNs are the coexistence of two adjoining populations of SCs with different proliferation rates (Barlow, 1978, 1997; Jiang and Feldman, 2005; Li and Clevers, 2010) and also the dual role of SCs that can act as organizers and also maintain their progeny undifferentiated (van den Berg et al., 1997; Pardo-Saganta et al., 2015). The functional meaning of SCs acting both as the organizer of the SCN and as SCs, as is the case for the QC in the root SCN, is possibly related to the self-organizing properties of the SCNs and the dynamic regulation of its size at the organ level. Furthermore, the existence of a population of SCs with different division rates results in the preservation of this population of cells for longer times, protecting them against deleterious mutations that otherwise might spread to the whole tissue (Clowes, 1956; Scadden, 2006). The root SCN is a well-described niche at the anatomical level, and we have a good understanding of the regulatory networks that underlie the acquisition of cell identity and hormonal profiles. Thus, the root SCN is a model system to describe the constraints of hormonal regulation of SCs activity that will then be instrumental to understand how the same may be occurring in other systems. The conceptual framework we presented in this review constitutes an important step toward this goal.

## Author Contributions

All authors listed have made a substantial, direct and intellectual contribution to the work, and approved it for publication.

## Conflict of Interest

The authors declare that the research was conducted in the absence of any commercial or financial relationships that could be construed as a potential conflict of interest.
